# Association of lactate to albumin ratio with the severity and prognosis of patients with sepsis admitted to the emergency intensive care unit: A prospective cohort study

**DOI:** 10.1097/MD.0000000000047064

**Published:** 2026-01-09

**Authors:** Xue Liu, Ling-Xiao Pang, Yao-Yao Li, Ying-Wei Ou, Rong-Cheng An, Yi-Fan Xu, Qian Li

**Affiliations:** a The Second Clinical Medical College, Hangzhou Normal University, Hangzhou, China; b Department of Emergency Medicine, Emergency and Critical Care Center, Zhejiang Provincial People’s Hospital (Affiliated People’s Hospital, Hangzhou Medical College), Hangzhou, China.

**Keywords:** albumin, kidney, lactic acid, morbidity, septic shock

## Abstract

The aim of this study is to evaluate the prognostic value of the lactate to albumin ratio (LAR) in predicting morbidity, acute kidney injury associated with sepsis (SA-AKI) and mortality in sepsis patients. This was a single-center prospective cohort study. All adult patients above the age of 18 with a diagnosis of sepsis who presented between January 1, 2024, and June 1, 2025, were included. The primary outcome was 28-day mortality, septic shock and SA-AKI. The patients were divided into non-shock group and shock group, non-acute kidney injury group and acute kidney injury group, survival group and death group for comparison. Group differences were significant based on LAR quartiles. Univariate and Logistic regression analyses showed that LAR was an independent risk factor for septic shock, septic acute kidney injury, and 28-day mortality. Elevated LAR stratification was associated with a significantly increased risk of shock, acute kidney injury, and 28-day mortality. LAR remained an independent risk factor for shock, acute kidney injury, and death when used as a continuous variable. Receiver operating characteristic (ROC) curve showed that the area under the curve (AUC) of septic shock was 0.705 (95% CI = 0.611–0.800) when the cutoff value of LAR was 0.106. When the cutoff value was 0.097, the AUC of sepsis-induced acute kidney injury was 0.762 (95% CI = 0.669–0.854). When the cutoff value was 0.098, the AUC of 28-day mortality was 0.863 (95% CI = 0.796–0.931). As the quartile of LAR layers increases, the risks of septic shock, SA-AKI and death gradually increased significantly. Early LAR has a certain predictive value for septic shock, SA-AKI and death outcomes.

## 1. Introduction

As a major global public health challenge, sepsis causes about 1.1 million deaths each year, accounting for 20% of the total number of deaths in the world.^[1]^ Its early identification and risk stratification directly determine the clinical prognosis. Despite the update of clinical guidelines in recent years, the in-hospital mortality of sepsis is still as high as 17 to 22%,^[2]^ and the mortality of patients with severe sepsis (such as multiple organ failure) can climb to more than 30%.^[3]^ However, the kidney is often involved in sepsis, leading to the occurrence of sepsis-associated acute kidney injury (SA-AKI). Approximately 30 to 50% of acute kidney injury (AKI) in the intensive care unit (ICU) is caused by sepsis. SA-AKI often leads to prolonged hospital stay, increased morbidity, and increased economic burden.^[4–6]^ At present, the prediction of mortality in sepsis is extremely difficult, mainly due to its high heterogeneity. It is not a single disease, but a uncontrolled reaction of the body caused by different pathogens and infection sites.^[7]^ Due to the great differences in age and underlying diseases of patients, the course of immune response and organ damage varies greatly. Its dynamic pathological evolution is both “inflammatory storm” and “immune paralysis,” which makes conventional biomarkers and scoring systems lag behind and are not accurate enough.^[8–10]^ Although emerging biomarkers such as PCT and sTREM-1 have certain predictive value, their application in resource-limited settings is limited due to high detection cost, long turnaround time, and equipment dependence.^[11,12]^

Lactate, a byproduct of anaerobic metabolism, accurately reflects the degree of cellular hypoxia and tissue hypoperfusion.^[13]^ However, lactate levels may increase in patients with liver dysfunction, or diabetes, making the prediction of lactate alone unreliable.^[14]^ As a key protein, serum albumin plays a significant role in maintaining blood osmolality balance, endothelial stability, and immune regulation, and it has been shown to predict poor prognosis in sepsis^[15]^ or AKI.^[16]^ However, factors such as nutritional status, inflammatory infection, and abnormal liver and kidney function can affect albumin levels, so the value of using albumin alone for prediction is limited.

Against this background, this research has focused on the lactic acid and the ratio of albumin (LAR). In recent years, a number of clinical studies have confirmed that LAR has significant value in the prognosis evaluation of sepsis. These studies have generally pointed out that LAR level on admission is an independent risk factor for mortality in patients with sepsis and that its predictive power is generally better than that of either lactate or albumin alone.^[17]^ In addition, increasing evidence also links high LAR to the risk of developing specific organ dysfunction such as sepsis-related AKI.^[18]^ As a regular screening program, admission blood can yield results after about 30 minutes to get the results, detection of low cost, and the index directly involved in systemic inflammatory response, kidney and other organ damage.^[19]^ Recent evidence has shown that this index is independently associated with the severity classification and 28-day prognosis of patients with sepsis. However, there are few prospective cohort studies on this index, and its prospects in clinical application need to be further explored.

The aim of this study was to evaluate the predictive performance of sepsis severity, SA-AKI, and 28-day mortality in a prospective cohort study, and to provide evidence for optimizing clinical risk stratification.

## 2. Materials and methods

### 2.1. Study population

As an exploratory, prospective cohort study, this study was designed to include all eligible patients seen at our single center during a defined feasibility period. The present study is an ongoing cohort study to evaluate the predictive value of LAR for mortality outcomes in patients with septic shock and SA-AKI. We conducted an observational study to analyze relevant clinical data and 28-day follow-up outcomes of patients with sepsis admitted to an EICU between January 2024 and July 2025. All eligible cases during the study period were included in the analysis, and no formal sample size calculation was performed beforehand.

The ethics committee of Zhejiang Provincial People’s Hospital approved the study protocol (No. 2024-072). Informed consent was obtained from patients or their families for all collected indicators.

Sepsis was defined according to the Sepsis-3 criteria as patients with a suspected infection and an acute increase in the Sequential Organ Failure Assessment (SOFA) score of ≥2 points. Septic shock was defined as a subset of sepsis in which profound circulatory, cellular, and metabolic abnormalities are present, characterized by persisting hypotension requiring vasopressors to maintain a mean arterial pressure (MAP) of ≥65 mm Hg and having a serum lactate level of > 2 mmol/L (18 mg/dL) despite adequate volume resuscitation. The diagnosis of SA-AKI was made based on the Kidney Disease: Improving Global Outcomes (KDIGO) criteria. Patients were classified as having SA-AKI if they met any of the following criteria within 48 hours of sepsis diagnosis: an increase in serum creatinine by ≥ 0.3 mg/dL (≥26.5μmol/L) within 48 hours; an increase in serum creatinine to ≥ 1.5 times baseline, which is known or presumed to have occurred within the prior 7 days; urine volume < 0.5 mL/kg/h for 6 hours.

Exclusion criteria were as follows: age < 18 years; cancer diseases; Immune system disease; organ transplantation or long-term use of glucocorticoids, immunosuppression, and chemoradiotherapy; the families declined to be included in the study; patients with renal failure and renal insufficiency who require renal replacement therapy; pregnancy or lactation; signed informed consent was not obtained.

### 2.2. Data collection

The clinical data of the enrolled patients were collected, including gender, age and white blood cell count (WBC), platelet count (PLT), D-D, B-type natriuretic peptide (BNP), the ratio of aspartate aminotransferase to alanine aminotransferase, urea nitrogen:creatinine (BUN:Scr), β-hydroxybutyric acid (β-OHB), lactate dehydrogenase (LDH), neutrophil count/lymphocyte count (N/L), procalcitonin (PCT), C-reactive protein (CRP), albumin (Alb), total bilirubin (TB), fibrinogen degradation products (FDP), lactic acid (Lac), partial thromboplastin time (APTT), acute physiology and chronic health evaluation II (APACHE II) score, inpatient nutritional risk screening NRS-2022 assessment scale (NRS score) within 24 hours of EICU admission, and 28-day prognosis of sepsis patients were followed up.

### 2.3. Statistical analysis

SPSS27.0 (IBM Corporation, Armonk) and GraphPadPrism 10.1.2 (GraphPad Software, San Diego) statistical software were used for data analysis. Shapiro–Wilk method was used to test the normality of measurement data, and Levene test was used to test the homogeneity of variance of data. Measurement data conforming to normal distribution were expressed as mean ± standard deviation (x ± s), and independent sample t test or corrected t test was used for comparison between the 2 groups. Measurements with skewed distribution were presented as medians and quartiles, and the rank-sum test was used for univariate comparisons between the 2 groups. Z values are 2-sided. The count data were expressed as example (rate), and the comparison between the 2 groups was analyzed by x^2^ test. LAR was analyzed and compared by quartile grouping, Binary Logistic regression was used to analyze the independent risk factors of sepsis patients with AKI, and the receiver operator characteristic curve was drawn. Receiver operating characteristic (ROC) curve was used to analyze the predictive value of each risk factor for the severity and prognosis of sepsis. *P* < .05 was considered statistically significant.

### 2.4. LAR analysis and outcomes

To explore whether there is a dose-response relationship between LAR and AKI risk, standard statistical analysis procedures were followed in this study. First, LAR was grouped by quartile to visually and robustly assess whether there was a linear association trend between LAR and AKI risk. After confirming the presence of an overall trend, further consideration was given to the inclusion of LAR as a continuous variable in the multivariate model for in-depth analysis, thus more precisely quantifying the change in AKI risk corresponding to each unit increase in LAR.

The primary outcome was all-cause mortality within 28 days, secondary outcomes were diagnoses of sepsis-induced AKI and septic shock.

## 3. Results

**Figure 1.**
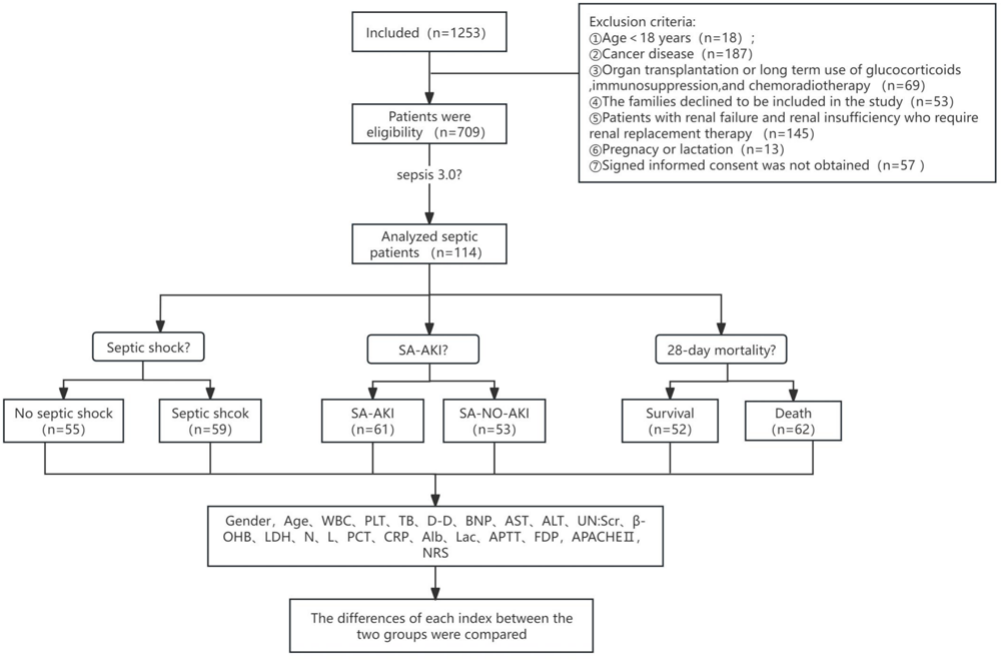
Study profile.

A total of 1253 patients admitted to EICU were observed from 2024 to 2025. After exclusion criteria, 709 patients were initially included in the study. After observation, patients with immune diseases and lost to follow-up were excluded, and a total of 114 septic patients were included in the final analysis. The analysis process is as shown in Figure 1. According to the quartile method, LAR was divided into 4 groups (Table 1).

**Table 1:** LAR quartiles were grouped.

LAR quartiles	Quartile	Septic shock	No septic shock	SA-AKI	SA-NO-AKI	Survival	Death
Quartile_1	<0.0486256	7	20	12	16	23	4
Quartile_2	0.0486256–0.0967305	15	15	4	25	20	9
Quartile_3	0.09673051–0.190972	15	14	18	11	8	21
Quartile_4	>0.190972	22	6	27	1	1	28

LAR = lactate to albumin ratio, SA-AKI = acute kidney injury associated with sepsis, SA-NO-AKI = sepsis not acute kidney injury.

### 3.1. Analysis the septic shock and no septic shock groups

Comparison of patients with septic shock and patients without septic shock: 59 patients with septic shock (51.75%) met the inclusion criteria, and 55 patients with non-septic shock (48.25%) were included in the control group. By single factor (Table 2), binary logistic regression results showed (Table 3) that LAR group, BUN:Scr, and WBC were independent risk factors for septic shock, and the higher LAR group was more likely to suffer from septic shock (Table 4).

**Table 2:** Univariate analysis of the variables of the groups of septic shock and no septic shock.

Variable	Septic shock (n = 59)	No septic shock (n = 55)	χ^2^/*t*/*z*	*P*
Gender				
Male	n = 38 (64.41%)	n = 39 (70.91%)	0.549	.295
Female	n = 21 (35.59%)	n = 16 (29.09%)		
Age	76.00 (66.00, 84.00)	72.00 (63.00, 81.00)	1.015	.310
LAR quartiles				
LAR-quartile 1	n = 7 (25.93%)	n = 20 (74.07%)	15.315	.002
LAR-quartile 2	n = 15 (50.00%)	n = 15 (50.00%)		
LAR-quartile 3	n = 15 (51.72%)	n = 14 (48.28%)		
LAR-quartile 4	n = 22 (78.57%)	n = 6 (21.43%)		
LAR group	3.00 (2.00, 4.00)	2.00 (1.00.4.00)	3.174	<.001
LAR	0.13 (0.07, 0.23)	0.07 (0.04, 0.12)	3.780	<.001
N/L (%)	15.50 (10.90, 34.43)	14.06 (9.04, 24.91)	1.231	.218
AST:ALT	1.91 (1.09, 2.86)	1.58 (1.21, 2.38)	0.811	.059
BUN:Scr	28.29 (19.67, 41.46)	22.95 (17.69, 36.44)	2.172	.030
β-OHB (mmol/L)	0.21 (0.10, 0.62)	0.22 (0.12, 0.56)	0.147	.883
LDH (U/L)	280.00 (222.00, 427.00)	269.00 (216.00, 361.00)	0.689	.491
D-D (µg/L)	5790.00 (2660.00, 12,580.00)	4200.00 (1700.00, 10,630.00)	0.953	.341
FDP (µg/L)	22,000.00 (8800.00, 47,700.00)	17,800.00 (7200.00, 36,500.00)	0.808	.419
PCT (ng/mL)	4.60 (1.00, 22.00)	1.90 (0.37, 11.00)	1.767	.077
TB (µmol/L)	13.60 (8.80, 28.80)	17.10 (11.70, 22.50)	1.177	.239
CRP (mg/L)	88.70 (51.70, 207.70)	76.20 (19.80, 164.40)	1.891	.059
WBC (×109)	12.07 (7.94, 20.22)	10.08 (7.87, 13.62)	2.229	.026
PLT (×109/L)	112.00 (74.00, 199.00)	134.00 (78.00, 176.00)	0.020	.984
BNP (pg/mL)	387.20 (111.00, 1276.40)	205.70 (57.90, 593.10)	1.843	.065
APTT (s)	31.30 (26.80, 37.60)	29.50 (26.00, 53.90)	1.208	.227
APACHEII score	21.00 (16.00, 27.00)	20.00 (15.00, 24.00)	0.860	.390
NRS score	4.00 (3.00, 6.00)	4.00 (3.00, 5.00)	1.450	.147

Data are shown as the mean ± SD and M (IQR).

Alb = albumin, APACHE II = acute physiology and chronic health evaluation II score, APTT = partial thromboplastin time, AST:ALT = the ratio of aspartate aminotransferase to alanine aminotransferase, BNP = B-type natriuretic peptide, BUN:Scr = urea nitrogen: creatinine, CRP = C-reactive protein, FDP = fibrinogen degradation products, IQR = interquartile range, Lac = Lactic acid, LAR = lactate to albumin ratio, LDH = lactate dehydrogenase, M = median, N/L = Neutrophil count/lymphocyte count, NRS score = inpatient nutritional risk screening NRS-2022 assessment scale, PCT = procalcitonin, PLT = platelet count, SD = standard deviation, TB = total bilirubin, WBC = white blood cell count, β-OHB = β-hydroxybutyric acid.

**Table 3:** Multivariate logistic regression of the significant variables in the groups of septic shock and no septic shock about LAR quartiles.

Variable	β	SE	Wald	*P*-value	OR	95% CI
LAR-quartile 2	0.846	0.592	2.042	.153	2.331	(0.730–7.440)
LAR-quartile 3	0.777	0.609	1.630	.202	2.175	(0.660–7.173)
LAR-quartile 4	2.070	0.654	10.027	.002	7.925	(2.201–28.541)
BUN:Scr	0.034	0.018	3.850	.050	1.035	(1.0000–1.071)
WBC	0.067	0.033	4.067	.044	1.069	(1.022–1.141)

95% CI = confidence interval, BUN:Scr = urea nitrogen: creatinine, LAR = the ratio of lactate to albumin, PLT = platelet count, OR = odds ratio, SE = standard error around the co-efficient, Wald = Wald chi-square test value, WBC = white blood cell count, β = co-efficient for the constant in the null model.

**Table 4:** Multivariate logistic regression of the significant variables in the groups of septic shock and no septic shock about continuity of LAR group.

Variable	β	SE	Wald	*P*_value	OR	95% CI
LAR group	0.592	0.800	16.084	.003	1.808	(1.228–2.660)
BUN:Scr	0.033	0.018	3.565	.059	1.034	(0.999–1.070)
WBC	0.070	0.033	4.419	.036	1.072	(1.005–1.145)

95% CI = confidence interval, BUN:Scr = urea nitrogen: creatinine, LAR = the ratio of lactate to albumin, OR = odds ratio, PLT = platelet count, SE = standard error around the co-efficient, Wald = Wald chi-square test value, WBC = white blood cell count, β = co-efficient for the constant in the null model.

**Figure 2.**
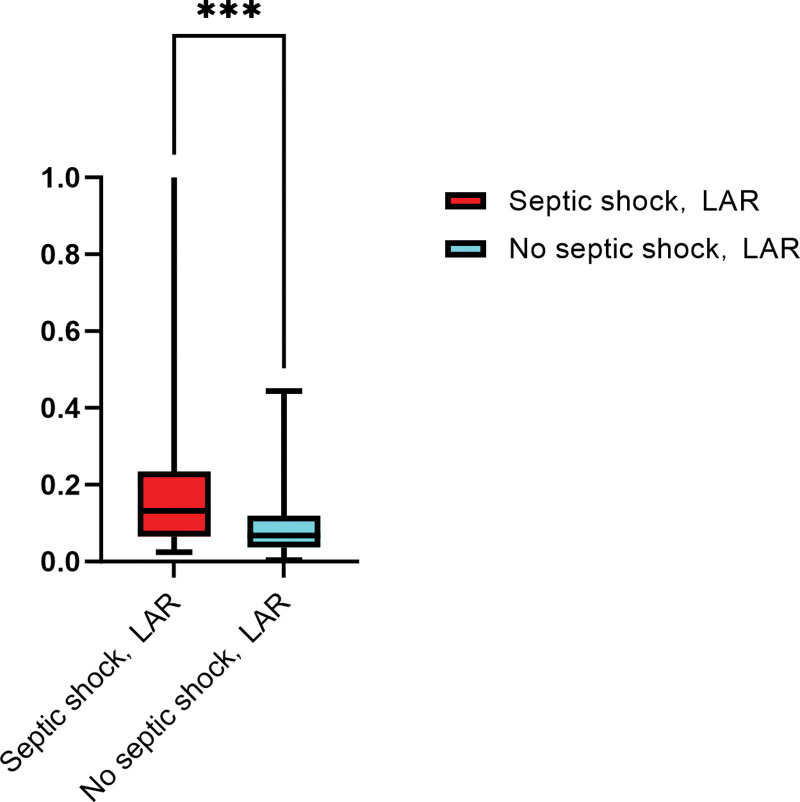
The comparison of septic shock and no septic shock groups about LAR. LAR = lactate to albumin ratio.

**Figure 3.**
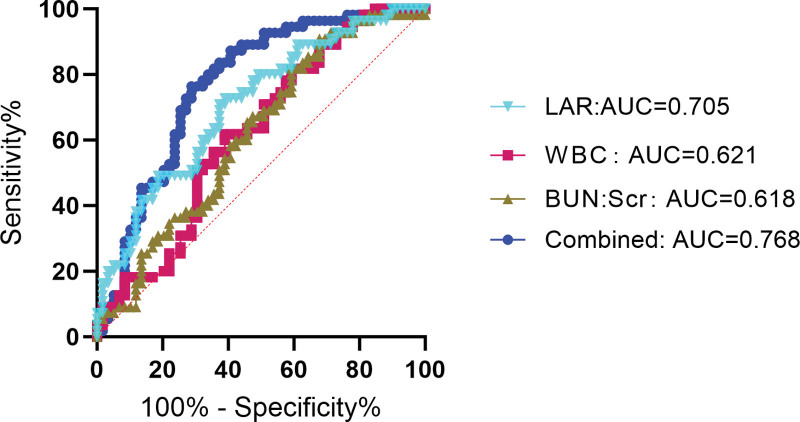
ROC curves of independent risk factors to distinguish septic shock patients. ROC = receiver operating characteristic.

When LAR as a continuous variable analysis, has a significant difference between the 2 groups (Fig. 2) and was independent risk factor for septic shock (Table 5). The results of ROC curve analysis showed (Table 6; Fig. 3) that WBC, BUN:Scr, and LAR had certain predictive value for patients with septic shock (all *P* < .05). When the cutoff value of BUN:Scr was 35.780, the area under the curve (AUC) was 0.618 (95% CI = 0.515–0.721); When the cutoff value of LAR was 0.106, the AUC was 0.705 (95% CI = 0.611–0.800). When the cutoff value of WBC was 11.550, the AUC was 0.621 (95% CI = 0.518–0.724); The cutoff value of the combined group was 0.523, and the AUC was 0.756 (95% CI = 0.664–0.848). Thus, it can be concluded that LAR has better prediction performance of septic shock than WBC and BUN:Scr, but the combination of the 3 has better prediction performance.

**Table 5:** Multivariate logistic regression of the significant variables in the groups of septic shock and no septic shock groups about LAR.

Variable	β	SE	Wald	*P*-value	OR	95% CI
LAR	5.217	0.018	5.243	0.022	184.425	(2.120–16,044.991)
BUN:Scr	0.040	0.018	5.129	0.024	1.041	(1.005–1.078)
WBC	0.067	0.034	3.983	0.046	1.069	(1.001–1.142)

95% CI = confidence interval, BUN:Scr = urea nitrogen: creatinine, LAR = the ratio of Lactate to Albumin, OR = odds ratio, PLT = platelet count, SE = standard error around the co-efficient, Wald = Wald chi-square test value, WBC = white blood cell count, β = efficient for the constant in the null model.

**Table 6:** The predictive value of each independent risk factors in patients with septic shock.

Variable	AUC	95% CI	*P*-value	cutoff value	Sensitivity (%)	Specificity (%)	Youden index
LAR	0.705	0.611–0.800	<.001	0.106	61.000	72.700	0.337
BUN:Scr	0.618	0.515–0.721	.030	35.780	32.200	90.900	0.231
WBC	0.621	0.518–0.724	.026	11.550	61.000	61.800	0.228
Combined	0.756	0.664–0.848	<.001	0.523	69.500	85.500	0.550

95% CI = confidence interval, AUC = area under the curve, BUN:Scr = urea nitrogen: creatinine, LAR = the ratio of lactate to albumin, OR = odds ratio, SE = standard error around the co-efficient, Wald = Wald chi-square test value, WBC = white blood cell count, β = co-efficient for the constant in the null model.

### 3.2. Analysis the SA-AKI and SA-NO-AKI groups

Comparison of patients with SA-AKI and patients with sepsis not acute kidney injury: 61 patients with SA-AKI (53.51%) met the inclusion criteria, and 53 patients with sepsis not acute kidney injury (46.49%) were included in the control group. By single factor (Table 7), binary logistic regression results showed (Table 8) that LAR group and APACHE II score were independent risk factors for SA-AKI, and the higher LAR group was more likely to suffer from SA-AKI (Table 9).

**Table 7:** Univariate analysis of the variables of the groups of SA-AKI and SA-NO-AKI.

Variable	SA-AKI (n = 61)	SA-NO-AKI (n = 53)	χ^2^/*T*/*Z*	*P*-value
Gender				
Male	n = 40 (65.57%)	n = 37 (69.81%)	0.232	.630
Female	n = 21 (34.43%)	n = 16 (30.19%)		
Age	76.75 ± 13.79	66.26 ± 16.94	3.690	<.001
LAR quartiles				
LAR-quartile 1	n = 12 (42.86%)	n = 16 (57.14%)	41.253	<.001
LAR-quartile 2	n = 4 (13.79%)	n = 25 (86.21%)		
LAR-quartile 3	n = 18 (62.07%)	n = 11 (37.93%)		
LAR-quartile 4	n = 27 (96.43%)	n = 1 (3.57%)		
LAR group	3.00 (2.00, 4.00)	2.00 (1.00, 2.00)	−4.976	<.001
LAR	0.17 (0.08, 0.25)	0.07 (0.04, 0.10)	−4.086	<.001
N/L (%)	15.40 (9.41, 39.67)	14.15 (8.68, 24.97)	−1.392	.164
AST: ALT	1.91 (1.22, 2.69)	1.15 (1.12, 2.44)	−1.226	.224
BUN: Scr	26.09 (20.01, 35.04)	24.52 (17.66, 33.08)	−0.855	.393
β-OHB (mmol/L)	0.26 (0.14, 0.60)	0.14 (0.09, 0.55)	−1.867	.062
LDH (U/L)	300.00 (224.00, 423.00)	265.0 (217.00, 351.00)	−1.653	.098
D-D (μg/L)	6070.00 (3790.00, 12,390.00)	4060.00 (1510.00, 8845.00)	−2.338	.019
FDP (μg/L)	23,600.00 (11,400.00, 45,750.00)	12,100.00 (7050.00, 39,750.00)	−1.829	.067
PCT (ng/mL)	8.60 (1.50, 22.50)	1.30 (0.32, 7.85)	−2.934	.003
TB (umol/L)	18.00 (9.70, 33.40)	14.70 (8.95, 18.55)	−2.082	.037
CRP (mg/L)	119.30 (51.60, 208.20)	75.90 (34.20, 134.00)	−2.233	.026
WBC (×10^9^)	11.92 (8.04, 18.04)	11.29 (8.03, 14.68)	−0.688	.504
PLT (×10^9^/L)	109.00 (69.00, 165.00)	144.00 (80.50, 200.50)	−1.846	.065
BNP (pg/mL)	429.5 (112.25, 1199.90)	187.40 (57.85, 554.90)	−2.034	.042
APTTs	40.80 (34.20, 55.68)	34.40 (29.30, 65.42)	−3.119	.002
APACHEII score	24.36 ± 8.62	18.09 ± 7.69	4.070	<.001
NRS score	6.00 (5.00, 7.00)	4.50 (4.00, 6.00)	−2.342	.019

Data are shown as the mean ± SD and M (IQR).

Alb = albumin, APACHE II = acute physiology and chronic health evaluation II score, APTT = partial thromboplastin time, AST:ALT = the ratio of aspartate aminotransferase to alanine aminotransferase, BNP = B-type natriuretic peptide, BUN:Scr = UREA nitrogen: creatinine, CRP = C-reactive protein, FDP = Fibrinogen degradation products, IQR = interquartile range, Lac = Lactic acid, LDH = lactate dehydrogenase, M = median, N/L = neutrophil count/lymphocyte count, NRS score = inpatient nutritional risk screening NRS-2022 assessment scale, PCT = procalcitonin, PLT = platelet count, SA-AKI = acute kidney injury associated with sepsis, SA-NO-AKI = sepsis not acute kidney injury, SD = standard deviation, TB = total bilirubin, WBC = white blood cell count, β-OHB = β-hydroxybutyric acid.

**Table 8:** Multivariate logistic regression of the significant variables in the groups of SA-AKI about LAR quartiles.

Variable	β	SE	Wald	OR	*P*-value	95% CI
Age	0.024	0.018	1.684	1.024	0.194	0.988–1.062
D-D	0.000	0.000	0.046	1.000	0.829	1.000–1.000
LAR-quartile 2	-2.404	0.893	7.251	0.090	0.007	0.016–0.520
LAR-quartile 3	0.630	0.670	0.883	1.877	0.348	0.504–6.987
LAR-quartile 4	3.831	1.227	9.756	46.124	0.002	4.167–510.560
TB	0.007	0.005	2.119	1.007	0.145	0.997–1.017
PCT	-0.017	0.016	1.127	0.983	0.288	0.952–1.015
CRP	0.005	0.004	1.385	1.005	0.239	0.997–1.014
APTT	-0.015	0.015	0.969	0.985	0.325	0.957–1.015
BNP	0.000	0.000	0.294	1.000	0.588	0.999–1.001
APACHEII score	0.121	0.040	9.388	1.129	0.002	1.045–1.229

95% CI = confidence interval, APACHE II = acute physiology and chronic health evaluation II score, APTT = partial thromboplastin time, BNP = B-type natriuretic peptide, CRP = C-reactive protein, LAR = the ratio of Lactate to Albumin, OR = odds ratio, PCT = procalcitonin, SA-AKI = acute kidney injury associated with sepsis, SE = standard error around the co-efficient, Wald = Wald chi-square test value, β = co-efficient for the constant in the null model.

**Table 9:** Multivariate logistic regression of the significant variables in the groups of SA-AKI and about LAR group.

Variable	β	SE	Wald	OR	*P*-value	95% CI
Age	0.030	0.016	3.407	1.031	.065	0.998–1.065
D-D	0.000	0.000	0.031	1.000	.859	1.000–1.000
LAR group	0.972	0.245	15.788	2.644	<.001	1.637–4.271
TB	0.002	0.004	0.300	1.002	.584	0.994–1.010
PCT	-0.010	0.013	0.683	0.990	.409	0.966–1.014
CRP	0.005	0.004	1.905	1.005	.168	0.998–1.012
APTT	-0.008	0.011	0.494	0.992	.482	0.972–1.014
BNP	0.000	0.000	1.353	1.000	.245	1.000–1.000
APACHE II	0.069	0.033	4.442	1.071	.035	1.005–1.142

95% CI = confidence interval, APACHE II = acute physiology and chronic health evaluation II score, APTT = partial thromboplastin time, BNP = B-type natriuretic peptide, CRP = C-reactive protein, LAR = the ratio of Lactate to Albumin, OR = odds ratio, PCT = procalcitonin, SA-AKI = acute kidney injury associated with sepsis, SE = standard error around the co-efficient, TB = Total bilirubin, Wald = Wald chi-square test value, β = co-efficient for the constant in the null model.

**Figure 4.**
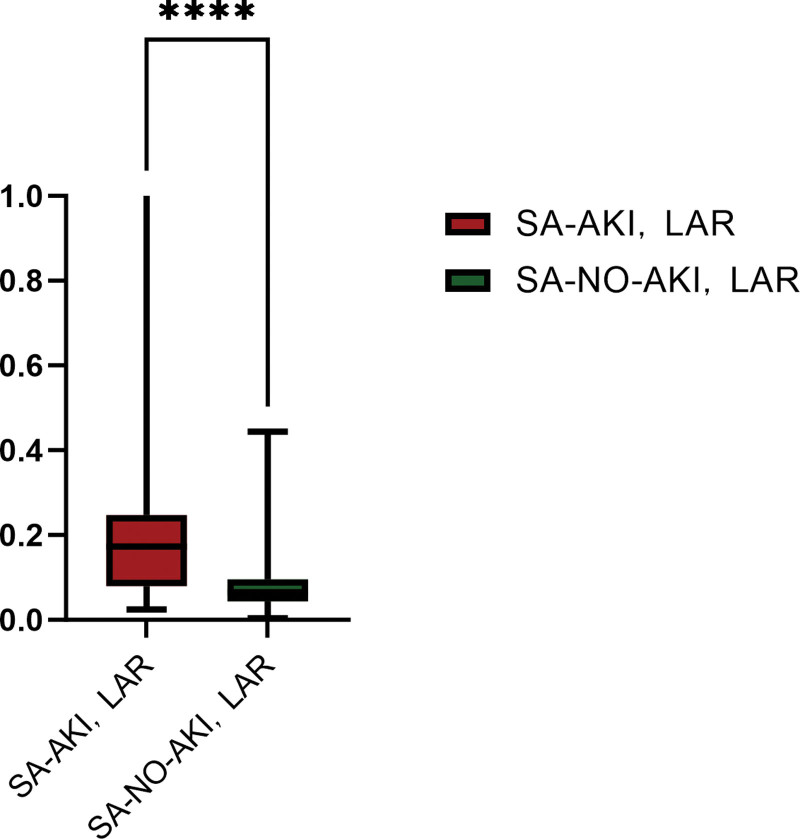
The comparison of SA-AKI and SA-NO-AKI groups about LAR. LAR = lactate to albumin ratio, SA-AKI = acute kidney injury associated with sepsis, SA-NO-AKI = sepsis not acute kidney injury.

**Figure 5.**
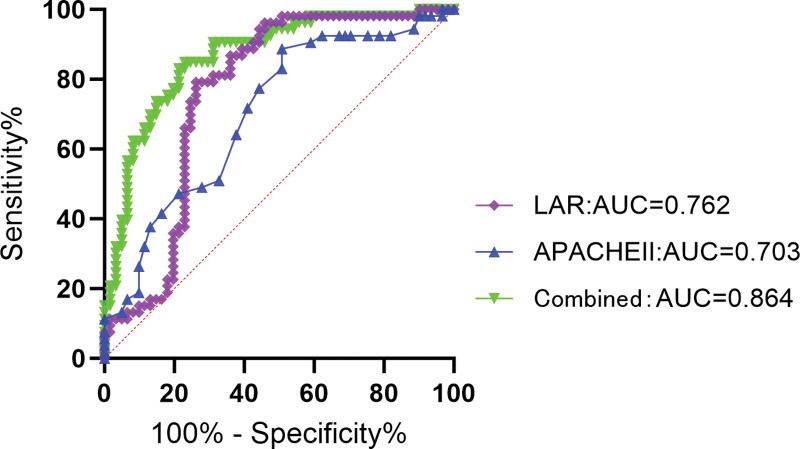
ROC curves of independent risk factors to distinguish SA-AKI and SA-NO-AKI. ROC = receiver operating characteristic, SA-AKI = acute kidney injury associated with sepsis, SA-NO-AKI = sepsis not acute kidney injury.

When LAR as a continuous variable analysis, has a significant difference between the 2 groups (Fig. 4) and was a independent risk factors for SA-AKI (Table 10). The results of ROC curve analysis showed (Table 11; Fig. 5) that APACHE II score and LAR had certain predictive value for patients with septic shock (all *P* < .05). When the cutoff value of APACHE II score was 24.500, the AUC was 0.703 (95% CI = 0.607–0.799); When the cutoff value of LAR was 0.097, the AUC was 0.762 (95% CI = 0.669–0.854); The cutoff value of the combined group was 0.543, and the AUC was 0.864 (95% CI = 0.796–0.932). Thus, it can be concluded that LAR has better prediction performance of SA-AKI than APACHE II score, but the combination of the 2 has better prediction performance.

**Table 10:** Multivariate logistic regression of the significant variables in the groups of SA-AKI about LAR.

Variable	β	SE	Wald	OR	*P*-value	95% CI
Age	0.032	0.017	3.578	1.032	.059	0.999–1.067
D-D	0.000	0.000	0.005	1.000	.946	1.000–1.000
LAR	12.466	3.791	10.780	254,214.965	.001	150.841–428,433,980.300
TB	0.003	0.004	0.548	1.003	.459	0.995–1.011
PCT	0.009	0.013	0.567	0.991	.451	0.967–1.015
CRP	0.004	0.004	1.266	1.004	.261	0.997–1.011
APTT	-0.010	0.011	0.741	0.990	.389	0.969–1.013
BNP	0.000	0.000	1.019	1.000	.313	1.000–1.001
APACHE II	0.070	0.033	4.597	1.073	.032	1.006–1.144

95% CI = confidence interval, APACHE II = acute physiology and chronic health evaluation II score, APTT = partial thromboplastin time, BNP = B-type natriuretic peptide, CRP = C-reactive protein, LAR = the ratio of Lactate to Albumin, OR = odds ratio, PCT = procalcitonin, SA-AKI = acute kidney injury associated with sepsis, SE = standard error around the co-efficient, TB = Total bilirubin, Wald = Wald chi-square test value, β = co-efficient for the constant in the null model.

**Table 11:** The predictive value of each independent risk factor in patients of SA-AKI.

Variable	AUC	95% CI	P-value	cutoff value	Sensitivity (%)	Specificity (%)	Youden index
APACHE II	0.703	0.607–0.799	<.001	24.500	49.200	88.700	0.379
LAR	0.762	0.669–0.854	<.001	0.097	73.800	79.200	0.530
Combined	0.864	0.796–0.932	<.001	0.543	77.000	84.900	0.619

95% CI = confidence interval, APACHE II = acute physiology and chronic health evaluation, AUC = area under the curve, LAR = the ratio of lactate to albumin, SA-AKI = acute kidney injury associated with sepsis.

### 3.3. Analysis the survival and death of septic patients

Comparison of patients of survival and death: 62 patients of death (54.39%) met the inclusion criteria, and 52 patients of survival (45.61%) were included in the control group. By single factor (Table 12), binary logistic regression results showed (Table 13) that LAR group and APACHE II score were independent risk factors for SA-AKI, and the higher LAR group was more likely to suffer from SA-AKI (Table 14).

**Table 12:** Univariate analysis of the variables of the groups of survival and death.

Variable	Survival (n = 52)	Death (n = 62)	χ^2^/*T*/*Z*	*P*
Gender				
Male	n = 37 (71.15%)	n = 40 (64.52%)	0.568	.451
Female	n = 15 (28.85%)	n = 22 (35.48%)		
Age	69.50 (55.00, 78.25)	77.50 (68.75, 87.25)	3.460	<.001
LAR quartiles				
LAR-quartile 1	n = 23 (85.19%)	n = 4 (14.81%)	48.00	.001
LAR-quartile 2	n = 20 (68.97%)	n = 9 (31.03%)		
LAR-quartile 3	n = 8 (27.59%)	n = 21 (72.41%)		
LAR-quartile 4	n = 1 (3.44%)	n = 28 (96.56%)		
LAR group	2.00 (1.00, 2.00)	3.00 (3.00, 4.00)	6.816	<.001
LAR	0.06 (0.04, 0.08)	0.17 (0.10, 0.24)	6.662	<.001
N/L (%)	14.04 (7.95, 23.00)	16.21 (11.17, 38.92)	2.088	.037
AST:ALT	1.49 (1.13, 2.51)	1.85 (1.18, 2.71)	1.198	.231
BUN:Scr	22.37 (16.23, 31.57)	27.12 (20.39, 38.14)	2.426	.015
β-OHB (mmol/L)	0.21 (0.09, 0.52)	0.24 (0.10, 0.59)	0.569	.569
LDH (U/L)	267.00 (223.25, 344.50)	300.00 (218.00, 444.00)	1.283	.200
D-D (µg/L)	4055.00 (1647.50, 8640.00)	6730.00 (3482.50, 15,525.00)	2.387	.017
FDP (µg/L)	12,150.00 (7025.00, 37,367.50)	24,100.00 (9475.00, 47,700.00)	2.119	.034
PCT (ng/mL)	1.60 (0.38, 7.43)	8.80 (0.71, 28.00)	2.762	.006
TB (µmol/L)	14.45 (9.10, 18.45)	17.70 (9.75, 28.80)	1.610	.107
CRP (mg/L)	74.35 (33.40, 131.25)	122.55 (51.58, 208.15)	2.384	.017
WBC (×109)	10.62 (8.12, 14.15)	11.92 (7.89, 18.61)	0.848	.397
PLT (×109/L)	143.00 (98.50, 209.50)	109.50 (61.75, 162.50)	2.398	.016
BNP (pg/mL)	185.50 (55.63, 624.30)	416.35 (112.88, 1268.75)	2.327	.020
APTT (s)	29.35 (25.78, 34.38)	34.10 (28.80, 39.43)	2.287	.022
APACHE II	17.88 ± 7.04	24.44 ± 8.96	4.278	<.001
NRS score	4.00 (3.00, 4.00)	5.00 (4.00, 6.00)	3.056	.002

Data are shown as the mean ± SD and M (IQR).

Alb = albumin, APACHE II = acute physiology and chronic health evaluation II score, APTT = partial thromboplastin time, AST:ALT = the ratio of aspartate aminotransferase to alanine aminotransferase, BNP = B-type natriuretic peptide, BUN:Scr = urea nitrogen: creatinine, CRP = C-reactive protein, FDP = fibrinogen degradation products, IQR = interquartile range, Lac = lactic acid, LDH = lactate dehydrogenase, M = median, N/L = neutrophil count/lymphocyte count, NRS score = inpatient nutritional risk screening NRS-2022 assessment scale, PCT = procalcitonin, PLT = platelet count, SD = standard deviation, TB = total bilirubin, WBC = white blood cell count, β-OHB = β-hydroxybutyric acid.

**Table 13:** Multivariate logistic regression of the significant variables in the groups of death about LAR quartiles.

Variable	β	SE	Wald	*P*-value	OR	95% CI
LAR-quartile 2	0.636	0.838	0.576	.448	1.889	0.366–9.762
LAR-quartile 3	2.363	0.839	7.929	.005	10.618	2.050–54.985
LAR-quartile 4	4.830	1.301	13.789	<.001	125.182	9.782–1601.904
D-D	0.000	0.000	0.000	.993	1.000	1.000–1.000
FDP	0.000	0.000	0.039	.844	1.000	1.000–1.000
Age	0.034	0.022	2.311	.127	1.035	0.990–1.081
N/L	-0.003	0.007	0.248	.618	0.997	0.983–1.010
CRP	0.000	0.004	0.001	.970	1.000	0.991–1.008
BNP	0.000	0.000	1.155	.282	1.000	1.000–1.001
APTT	0.005	0.010	0.297	.586	0.995	0.976–1.014
PLT	-0.001	0.004	0.125	.724	0.999	0.990–1.007
NRS score	0.034	0.187	0.033	.855	0.966	0.669–1.395
BUN: Scr	0.037	0.025	2.136	.144	1.037	0.988–1.090
APACHE II	0.095	0.046	4.251	.039	1.009	1.005–1.203
PCT	0.022	0.013	3.087	.079	1.022	0.997–1.048

95% CI = confidence interval, APACHE II = acute physiology and chronic health evaluation II score, APTT = partial thromboplastin time, BNP = B-type natriuretic peptide, BUN:Scr = urea nitrogen:creatinine, CRP = C-reactive protein, FDP = Fibrinogen degradation products, LAR = the ratio of lactate to albumin, N/L = neutrophil count/lymphocyte count, NRS Score = inpatient nutritional risk screening NRS-2022 assessment scale, OR = odds ratio, PCT = procalcitonin, PLT = platelet count, SE = standard error around the co-efficient, Wald = Wald chi-square test value, β = co-efficient for the constant in the null model.

**Table 14:** Multivariate logistic regression of the significant variables in the groups of death about continuity of LAR group.

Variable	β	SE	Wald	*P*_value	OR	95% CI
LAR group	1.525	2.317	15.682	<.001	4.596	2.373–8.901
D-D	0.000	0.000	0.033	.857	1.000	1.000–1.000
FDP	0.000	0.000	0.166	.684	1.000	1.000–1.000
Age	0.038	0.022	2.915	.088	1.038	0.994–1.084
N/L	0.002	0.006	0.141	.708	0.988	0.986–1.009
CRP	0.001	0.004	0.021	.885	1.001	0.992–1.009
BNP	0.000	0.000	1.501	.220	1.000	1.000–1.001
APTT	-0.005	0.009	0.334	.563	0.995	0.977–1.013
PLT	-0.001	0.004	0.004	.784	0.999	0.991–1.007
NRS	0.084	0.185	0.208	.648	0.919	0.640–1.320
BUN:Scr	0.034	0.025	1.922	.166	1.035	0.986–1.086
APACHE II	0.092	0.046	4.076	.043	1.097	1.003–1.200
PCT	0.019	0.012	2.439	.118	1.019	0.995–1.043

95% CI = confidence interval, APACHE II = acute physiology and chronic health evaluation II score, APTT = partial thromboplastin time, BNP = B-type natriuretic peptide, BUN:Scr = urea nitrogen:creatinine, CRP = C-reactive protein, FDP = Fibrinogen degradation products, LAR = the ratio of lactate to albumin, N/L = neutrophil count/lymphocyte count, NRS Score = inpatient nutritional risk screening NRS-2022 assessment scale, OR = odds ratio, PCT = procalcitonin, PLT = platelet count, SE = standard error around the co-efficient, Wald = Wald chi-square test value, β = co-efficient for the constant in the null model.

**Figure 6.**
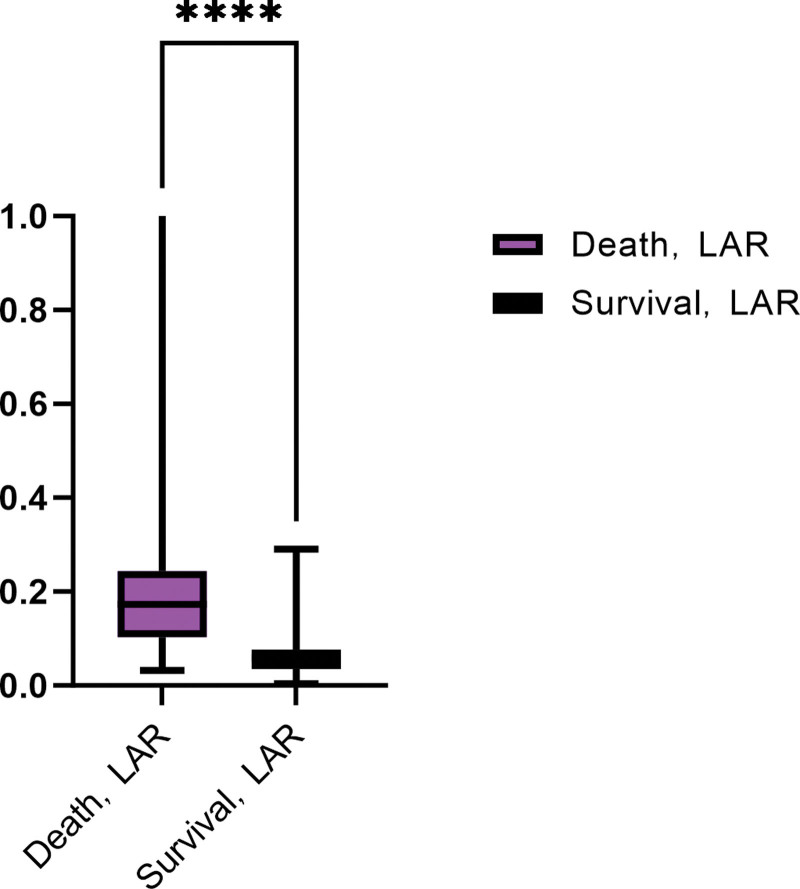
The comparison of Death and Survival groups about LAR. LAR = lactate to albumin ratio.

**Figure 7.**
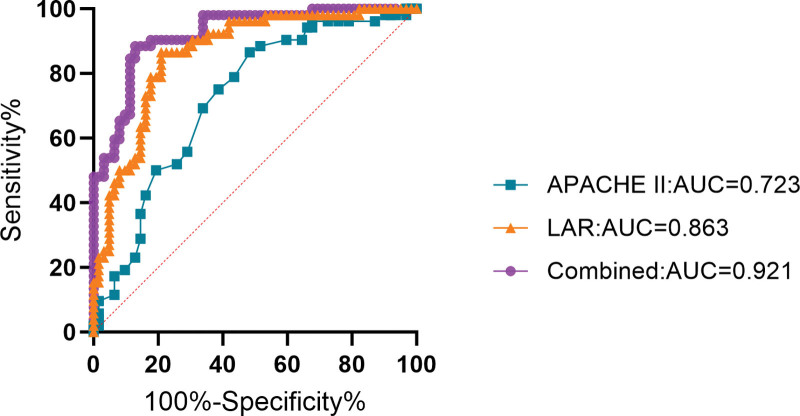
ROC curves of independent risk factors to distinguish death and survival. ROC = receiver operating characteristic.

When LAR as a continuous variable analysis, has a significant difference between the 2 groups (Fig. 6) and was a independent risk factors for SA-AKI (Table 15). The results of ROC curve analysis showed (Table 16; Fig. 7) that APACHE II score and LAR had certain predictive value for patients of death (all *P* < .05). When the cutoff value of APACHE II score was 23.500, the AUC was 0.723 (95% CI = 0.630–0.817); When the cutoff value of LAR was 0.098, the AUC was 0.863 (95% CI = 0.796–0.931); The cutoff value of the combined group was 0.499, and the AUC was 0.921 (95% CI = 0.873–0.969). Thus, it can be concluded that LAR has better prediction performance of death than APACHE II score, but the combination of the 2 has better prediction performance.

**Table 15:** Multivariate logistic regression of the significant variables in the groups of death about LAR.

Variable	β	SE	Wald	*P*-value	OR	95% CI
LAR	20.807	5.772	12.993	<.001	10,864	3267.749–8.903E + 13
D-D	0.000	0.000	0.003	.958	1.000	1.000–1.000
FDP	0.000	0.000	0.076	.783	1.000	1.000–1.000
Age	0.039	0.022	3.151	.076	1.040	0.996–1.086
N/L	-0.005	0.007	0.579	.447	0.995	0.981–1.008
CRP	0.001	0.004	0.086	.770	0.999	0.990–1.007
BNP	0.001	0.000	1.782	.182	1.001	1.000–1.001
APTT	-0.005	0.009	0.346	.557	0.995	0.977–1.013
PLT	0.000	0.004	0.002	.962	1.000	0.992–1.008
NRS score	0.072	0.177	0.165	.684	0.931	0.658–1.317
BUN: Scr	0.046	0.025	3.281	.070	1.047	0.996–1.101
APACHE II	0.089	0.044	4.145	.042	1.093	1.003–1.191
PCT	0.023	0.012	3.748	.053	1.023	1.000–1.001

95% CI = confidence interval, APACHE II = acute physiology and chronic health evaluation II score, APTT = partial thromboplastin time, BNP = B-type natriuretic peptide, BUN:Scr = urea nitrogen:creatinine, CRP = C-reactive protein, FDP = Fibrinogen degradation products, LAR = the ratio of lactate to albumin, N/L = neutrophil count/lymphocyte count, NRS Score = inpatient nutritional risk screening NRS-2022 assessment scale, OR = odds ratio, PCT = procalcitonin, PLT = platelet count, SE = standard error around the co-efficient, Wald = Wald chi-square test value, β = co-efficient for the constant in the null model.

**Table 16:** The predictive value of each independent risk factor in patients of death.

Variable	AUC	95% CI	*P*-value	cutoff value	Sensitivity (%)	Specificity (%)	Youden index
LAR	0.863	0.796–0.931	<.001	0.098	79.00	86.500	0.655
APACHE II	0.723	0.630–0.817	<.001	23.500	51.600	86.500	0.381
Combined	0.921	0.873–0.969	<.001	0.499	87.100	88.500	0.756

95% CI = confidence interval, APACHE II = acute physiology and chronic health evaluation, AUC = area under the curve, LAR = the ratio of lactate to albumin.

## 4. Discussion

The above research and data analysis have revealed that the LAR at the time of admission is an independent risk factor for the severity of the disease, the cumulative impact on the kidneys, and the 28-day mortality rate of sepsis patients. As the quartile of LAR layers increases, the risk of septic shock, AKI, and death for patients also increases, which is highly consistent with recent database studies.^[18,19]^ Patients with high LAR not only have a significantly increased risk of organ failure, but also have a several-fold increased risk of death compared with patients with low LAR. In addition, this conclusion is strongly supported by the landmark study.^[20,21]^ Cakir et al demonstrated that LAR (AUC = 0.869) was better at predicting in-hospital mortality than albumin (AUC = 0.812) and lactate (AUC = 0.816) separately.^[22]^ Shin et al reported that among 946 patients diagnosed with sepsis, LAR exhibited a better predictive capacity for 28-day mortality compared to lactate.^[20]^ However, the 3 LAR cutoff values that we identified in our current study were lower than those in other studies, possibly because we enrolled more patients with sepsis in an early or relatively mild state, when minor metabolic disturbances may trigger the pathophysiological process of renal injury or signal that significant injury is about to occur. Thus, the lower cutoff we identified may have more sensitively captured patients in “critical” or “early warning” status. This suggests that the predictive threshold of LAR is not static and needs to be interpreted in the context of specific clinical situations and patient characteristics.

From a pathophysiological perspective, the predictive value of the LAR stems from its dual reflection of the core mechanism of sepsis. On the one hand, elevated lactate is far from the result of Type A tissue hypoxia alone. In sepsis, “inflammatory storm”-driven aerobic glycolysis (the “Warburg effect”),^[23]^ catecholamine-stimulated Na^+^/K^+^-atpase activity, impaired hepatic clearance, and mitochondrial dysfunction (Type B) collectively contribute to hyperlactatemia.^[2,24,25]^ Its level profoundly reflects the severity of tissue malperfusion and cellular metabolic crisis. On the other hand, hypoalbuminemia is an important marker of sepsis. The mechanism of hypoalbuminemia is complex, including albumin extravasation (vascular leakage) caused by endothelial glycocalyx destruction by inflammatory factors (such as TNF-α and IL-6), inhibition of liver synthesis (preferentially synthesis of acute-phase proteins such as C-reactive protein), oxidative damage and accelerated decomposition of albumin. It also reflects the patient’s nutritional reserve and chronic health status. Thus, the LAR would combine “hit strength” (high lactate), which represents the severity of acute injury, with “hit resistance” (low albumin), which reflects physiological reserve, endothelial integrity, and nutritional status.^[26–28]^ A high ratio signals that the organism is simultaneously subjected to a violent acute attack and a weak defensive base, and necessarily points to worse clinical outcomes. Compared with SOFA score, q SOFA lactic acid detection or single traditional indicators, such as LAR ratio showed significant prediction.^[29]^ First, it has early warning value: lactate and albumin can be obtained quickly and routinely on admission, long before the complete 24-hour data on which SOFA scores typically rely.^[30]^ Second, it is cost-effective and widely accessible, with universal testing availability and low cost, especially suitable for resource-limited Settings (e.g., primary hospitals, emergency departments).^[21]^ Moreover, the LAR provides more comprehensive and specific information, avoiding the limitations of a single index.^[17]^ For example, isolated lactate elevations may result from nonhypoxic factors such as seizures, whereas isolated hypoalbuminemia may result from chronic liver disease.^[20]^ The LAR more specifically points to the multiple imbalance state of “metabolism-inflammation-reserve” peculiar to sepsis. Chen found that LAR was even better than SOFA score in predicting the risk of early deterioration in patients with sepsis.^[31]^

Another key finding is that the LAR cutoff values for predicting septic shock, SA-AKI, and death fell in the third LAR quartile. Given its excellent performance, LAR levels are promising for clinical management of sepsis. It can be used as a powerful risk stratification and rapid triage tool in the emergency department to identify high-risk patients with high LAR levels, prioritize their admission to the ICU, and initiate intensive treatment, which is consistent with and may complement the principles of early goal-directed therapy (EGDT).^[32]^ Patients with LAR values above a specific cutoff (i.e., classified as group 3 or 4) were identified as being in the “very high risk” group. For this group, more aggressive early intervention strategies, such as more frequent hemodynamic monitoring, earlier administration of broad-spectrum antibiotics, and preventive measures to protect renal function, should be initiated. Conversely, patients with LAR values below this cutoff (i.e., in the first or second group) are relatively in the “low-medium risk” group, which helps avoid overtreatment and thus optimistically allocate health care resources.

Limitations: in this study, the LAR cutoff values determined in our study were significantly lower than that reported in previous studies on large samples in databases.^[18,29]^ This may be due to the high heterogeneity of the sepsis patient population.^[33]^ There may be significant differences in baseline characteristics (such as age, underlying disease, source of infection, initial severity), definition criteria of septic shock, sample size, and clinical practice among different regions. In addition, the hospital in this study was A provincial hospital, and the sources of patients were diverse, such as after surgery, emergency department, transfer from other hospitals, etc. Many patients may have received clinical interventions (such as albumin infusion or renal replacement therapy) before admission to EICU, which may directly affect the LAR, and its effect needs to be more accurately evaluated in subsequent studies.

Future research should focus on the following aspects: conducting multi-center prospective cohort studies with large samples to verify its universality; Further exploration of individualized treatment strategies based on LAR level (such as setting the resuscitation plan with the goal of ratio change); LAR levels should be combined with other novel biomarkers (such as specific cytokines and endothelial injury markers) or artificial intelligence models to build more accurate prediction tools. To systematically study the quantitative association of LAR dynamic change patterns, such as descent slope, with prognosis.

## 5. Conclusion

In summary, LAR has shown some potential in predicting sepsis severity, involving AKI, and 28-day mortality, and in clinical practice, monitoring LAR can aid in treatment decision making, disease management, and prognostic assessment. Future multicenter prospective studies are warranted to investigate whether better control of LAR improves clinical outcomes.

## Author contributions

**Conceptualization:** Qian Li.

**Data curation:** Xue Liu, Ling-Xiao Pang, Ying-Wei Ou, Rong-Cheng An, Yi-Fan Xu.

**Formal analysis:** Qian Li, Xue Liu, Ling-Xiao Pang.

**Investigation:** Yao-Yao Li.

**Methodology:** Xue Liu, Qian Li.

**Project administration:** Qian Li, Ling-Xiao Pang.

**Validation:** Xue Liu.

**Writing – original draft:** Xue Liu.
